# Progressive high-fluence epithelium-on accelerated corneal crosslinking: a novel corneal photodynamic therapy for early progressive keratoconus

**DOI:** 10.3389/fmed.2023.1198246

**Published:** 2023-08-21

**Authors:** Cosimo Mazzotta, Anna Pandolfi, Marco Ferrise

**Affiliations:** ^1^Departmental Ophthalmology Unit, USL Toscana Sudest, Postgraduate Ophthalmology School, Alta Val d'Elsa Hospital, University of Siena, Siena, Italy; ^2^Siena Crosslinking Center, Siena, Italy; ^3^Civil and Environmental Engineering Department, Politecnico di Milano, Milan, Italy; ^4^Studio Oculistico Ferrise, Lamezia Terme, Italy

**Keywords:** keratoconus, corneal ectasia, corneal cross-linking, refractive surgery, Epi-On CXL

## Abstract

**Purpose:**

To assess the preliminary clinical results of a new, progressively higher fluence-pulsed light Epi-On accelerated crosslinking nomogram (PFPL M Epi-On ACXL) in the treatment of progressive keratoconus (KC).

**Setting:**

Siena Crosslinking Center, Siena, Italy.

**Methods:**

A prospective pilot open, non-randomized interventional study, including 32 eyes of 32 young-adult patients over 26 years old with Stages I-III progressive KC undergoing PFPL M Epi-On ACXL, was conducted. Riboflavin loading was performed by using Paracel I 0.25% for 4 min and Paracel II 0.22% for 6 min. The Avedro KXL System (Glaukos-Avedro, Burlington, USA) was used for pulsed-light accelerated crosslinking (ACXL) at air room 21% oxygenation and 13 min of UV-A irradiation. The treatment fluence was set at 7.2 J/cm^2^, 8.6 J/cm^2^, and 10.0 J/cm^2^ in corneas with baseline pachymetry <420 μm (group 1: 8 eyes), ≥ 420 μm <460 μm (group 2, 11 eyes), and ≥ 460 μm (group 3, 13 eyes), respectively. Uncorrected distance visual acuity (UDVA), best-spectacle corrected visual acuity (BSCVA), Scheimpflug corneal tomography, and anterior segment OCT (AS-OCT) data were collected at baseline and postoperatively at 1, 3, and 6 months.

**Results:**

UDVA and BSCVA improved in all groups (*P* ≤ 0.05). Maximum keratometry values (K max) showed a significant decrease in the 10.0 J/cm^2^ group (Δ −1.68 D). The coma (HOAs) value improved significantly by the sixth month in all groups. OCT average demarcation lines were 211 ± 19 μm in group 1, 245 ± 23 μm in group 2, and 267 ± 21 μm in group 3.

**Conclusions:**

The preliminary results show that pachymetry-based PFPL M Epi-On ACXL nomogram stabilizes ectasia progression. Higher fluence Epi-On ACXL increases CXL penetration, with better functional outcomes in the absence of complications.

## Introduction

To date, riboflavin–ultraviolet-A (UV-A) corneal collagen crosslinking (CXL) with epithelium removal (Epi-Off CXL) represents the gold standard of treatment for progressive keratoconus (KC) and other corneal ectasias (1). The main reason behind this is that the removal of the epithelial barrier allows for adequate diffusion of both riboflavin and oxygen into the corneal stroma, which is necessary to achieve an adequate photo-oxidative reaction and, consequent, the desired formation of collagen fibrils crosslinking with adjacent fibrils (1–3).

From a clinical and practical perspective, a safer and faster transepithelial crosslinking treatment with an efficacy profile comparable to Epi-Off CXL, with no adverse events and capable of quickly rehabilitating patients with progressive KC to resume their school or work activities, represents a great challenge and a highly interesting goal. In fact, there are several indisputable advantages to performing a corneal CXL whilst maintaining the epithelial layer. Some of the primary advantages are the elimination of the risk of infectious keratitis and persistent stromal haze, easier functional recovery for patients with minimal postoperative pain and discomfort, and the ability to avoid microstructural damage to the ocular surface so that patients may return to their normal activities the day after (4).

Another substantial advantage of Epi-On treatments is the possibility of performing the procedure fully in an outpatient modality, without the need for a sterile operating theater. This approach makes the clinical workflow more linear and is also cost-effective for the surgeon. In cases of bilateral progressive keratoconus (KC), a simultaneous bilateral treatment can also be performed, with a clear advantage for both the patient and the clinic in terms of organizational burden.

Unfortunately, many studies have shown that the initial adoption of the original 5.4 J/cm^2^ Epi-On corneal crosslinking has been largely unsuccessful due to a lack of ectasia stabilization. In 2012, Koppen et al.'s cohort study evaluated the efficacy of CXL treatment without epithelial debridement. The standard CXL irradiation was preceded by the instillation of proparacaine drops 0.5% preserved with BAC 0.005% every 5 min for 30 min. The results showed significant continuous maximum K increasing and thinnest point decreasing (KC progression) throughout the study in 100% of eyes (5). Similarly, in 2013, Caporossi et al.'s study in a population of pediatric and young adult patients treated with transepithelial (Epi-On) CXL showed that, after relative improvement in the first 3 to 6 months, the UDVA and CDVA gradually returned to baseline preoperative values. After 12 months of stability, K max and pachymetry values worsened at 24 months. Functional results after transepithelial CXL showed keratoconus instability, particularly in pediatric patients 18 years old and younger. There was also functional regression in patients between 19 years and 26 years old after 24 months of follow-up. In fact, 50% of pediatric patients were retreated with Epi-Off CXL due to significant deterioration of all parameters after 1 year of follow-up (6).

In 2016, Gatzioufas et al.'s prospective interventional multicentre study confirmed the clinical inefficacy of transepithelial CXL, documenting that after a 12-month follow-up, progression (defined by an increase in K max >1.00 diopter) still occurred in 46% of eyes (7).

Fortunately, everything has changed, and everything is changing from the original Epi-On CXL protocol. In recent years, the failure of the outset of Epi-On CXL has prompted researchers to utilize all the gathered clinical experience and deepen their understanding of the physiochemical mechanisms underlying crosslinking kinetics through multiple pathways. It has driven them to implement every small detail to bring the efficacy of transepithelial techniques ever closer to Epi-Off CXL (8).

Many parameters have been adapted to optimize the effectiveness of Epi-On accelerated crosslinking. The most important was represented by the increased fluence that passed from the original 5.4 to 7 J/cm^2^, which ensured greater penetration, visibility of deeper demarcation lines, and higher efficacy of the new Epi-On procedures without intraoperative supplemental oxygen (9).

The first international study on Epi-On customized crosslinking with intraoperative supplemental oxygen also demonstrated different depths of the demarcation line, according to a double fluence based on corneal curvature: 7.2 and 10 J/cm^2^, respectively (10).

The fact that fluence was a major player in the crosslinking procedure was clinically proven only in 2016 by Seiler et al. and Mazzotta et al. The authors used topography-guided accelerated crosslinking protocols with 7.2, 10, and 15 J/cm^2^ variable fluence in Epi-Off ACXL modality without adverse events for corneal stroma and endothelium (11, 12).

Another important parameter was the increased concentration of the chemically enhanced riboflavin solutions, now being used at 0.22 and 0.25% instead of 0.1%, which is significant considering that the penetration of riboflavin through the intact epithelium was more than halved compared with its passive diffusion after epithelium removal (13, 14).

An important method for improving riboflavin loading into the stroma through the epithelium *in situ* is the iontophoresis technique, which was recently renewed by Mazzotta et al. in the new iontophoresis protocol with enhanced fluence and pulsed light mode of UV-A irradiation (15, 16).

The use of pulsed UV-A light in a duty cycle of 1 s on 1 s off to partially enhance intraoperative oxygen diffusion and drive the aerobic photochemistry of the crosslinking reaction was based on lab demonstrations and on the literature showing a deeper stromal demarcation line when compared to the accelerated continuous light CXL protocol, with less microstructural damage and improved stromal oxygenation (17–20).

Recent laboratory studies have proven that higher fluences between 8 and 10 J/cm^2^ increase the biomechanical impact of CXL (21). The progressively higher fluence protocols enhance Epi-Off and Epi-On efficacy, as demonstrated in past and recent literature (9–12, 15). The increased fluence variance may be the key to optimizing the photodynamic process of Epi-On crosslinking, paving the way to the final switch (9). We present the preliminary data of the new pachymetry-based progressively higher fluence Epi-On nomogram as a new paradigm of corneal photodynamic therapy for early progressive KC (22–24).

## Materials and methods

A prospective pilot open, non-randomized interventional study, including 32 eyes of 32 young-adult patients over 26 years old with Stages I, II, and III progressive KC undergoing PFPL M Epi-On CXL, was conducted. The treatment protocol was approved by the Institutional Review Board of the Siena Crosslinking Center (Code: *PFPL.EPION 2.0*) under the tenets of the Helsinki Declaration, taking into account no expected risk of adverse events, as fluences between 5.4 and 15 J/cm^2^ have already been safely used and published in the literature for both Epi-Off and Epi-On customized accelerated CXL treatments (10–12). Likewise, riboflavin solutions (Paracel parts 1 and 2 by Glaukos-Avedro, Burlington, USA) already approved in the market for ACXL therapy of progressive KC were adopted (10–12).

The progression of KC was defined as an increase in K max ≥1 diopter (D) using the Scheimpflug–Placido corneal tomography system Sirius, Costruzione Strumenti Oftalmici (C.S.O.), Florence, Italy. It was also defined by a minimum corneal thickness (MCT) reduction ≥ 10 μm, worsening of uncorrected distance visual acuity (UDVA) and corrected distance visual acuity (CDVA) ≥ 0.1 decimal equivalent, or a change of ≥ 0.5 in the mean refractive spherical equivalent (MRSE) in the last 6 months of clinical and instrumental observation. MCT was required to be at least 400 μm (including epithelium). Patients with clear corneas with no sub-apical opacities or scars, no history of previous herpes simplex virus infection and other infectious keratitis or autoimmune diseases, and no severe dry eye were included.

All patients signed a specific informed consent form.

The demographic data of the study are displayed in Table 1.

**Table 1:** Demographic baseline data.

**Patients**	**32 (28 male patients/4 female patients)**
Group 1 (Fluence 7.2 J/cm^2^)	8 (pachymetry <420 μm)
Group 2 (Fluence 8.6 J/cm^2^)	11 (pachymetry ≥ 420 μm <460 μm)
Group 3 (Fluence 10 J/cm^2^)	13 (pachymetry ≥ 460 μm)
Eyes total	32
Age	27.9 y (range 26-31 y).
M/F ratio	28/4 (87.5% male)
UDVA (uncorrected distance visual acuity, *decimal equivalents*)	*Group 1*: 0.32 ± 0.07
	*Group 2*: 0.41 ± 0.08
	*Group 3*: 0.44 ± 0.11
BCDVA (best-corrected distance visual acuity, *decimal equivalents*)	*Group 1*: 0.63 ± 0.15
	*Group 2*: 0.77 ± 0.11
	*Group 3*: 0.75 ± 0.12
K max (maximum curvature simulated K reading, *dioptres*)	*Group 1*: 48.76 ± 0.9 D
	*Group 2*: 48.40 ± 0.4 D
	*Group 3*: 48.33 ± 0.6 D
COMA (high order aberration, *μm*)	*Group 1*: 0.68 ± 0.12
	*Group 2*: 0.69 ± 0.07
	*Group 3*: 0.64 ± 0.06
MCT (minimum corneal thickness, *μm*)	*Group 1*: 401 ± 11 μm
	*Group 2*: 442 ± 14 μm
	*Group 3*: 481 ± 15 μm
Follow-up	6 months

### Measurements and devices

Ophthalmic evaluations were performed before CXL and at all follow-up visits (1, 3, and 6 months). The evaluation included UDVA, best-spectacle corrected distance visual acuity (BCDVA), biomicroscopic corneal examination, ocular surface disease index (OSDI) test, and non-invasive topographic break-up time test (NI-BUT) for excluding dry-eye disease. Scheimpflug-based corneal tomography (Sirius, CSO, Florence, Italy) was used to measure the maximum curvature simulated K reading (K max), high-order aberration (coma), and MCT. Anterior segment optical coherence tomography (AS-OCT) was performed using the I-Vue (Optovue, Fremont CA, USA) to assess the demarcation line depth on the first postoperative month.

### Surgical procedure

The treatment parameters of PHFPL Epi-On ACXL are displayed in Table 2.

**Table 2:** Progressive Fluence Pulsed Light Epi-On accelerated crosslinking M nomogram, developed by Mazzotta and Ferrise at the Siena crosslinking center, Italy.

**Parameter**	**Variable**	**Variable**	**Variable**
Treatment target	KC stabilization	KC stabilization	KC stabilization
Thinnest point	<420 μ	≥ 420 μm <460 μm	≥ 460 μm
Fluence (total) (J/cm^2^)	7.2 J/cm^2^	8.6 J/cm^2^	10 J/cm^2^
Soak time and interval (min)	10 min (4 + 6)	10 min (4 + 6)	10 min (4 + 6)
Intensity (mW)	18 mW/cm^2^	22 mW/cm^2^	26 mW/cm^2^
Irradiation time	13 min	13 min	13 min
Epithelium status	On	On	On
Chromophore	Riboflavin	Riboflavin	Riboflavin
Chromophore carrier and concentration. Paracel part 1 (0.25%)	BAK, EDTA, trometamol	BAK, EDTA, trometamol	BAK, EDTA, trometamol
	HPMC NaCl	HPMC NaCl	HPMC NaCl
	Phosphate buffered saline solution	Phosphate buffered saline solution	Phosphate buffered saline solution
Chromophore carrier and concentration. Paracel part 2 (0.22%)	NaCl Phosphate buffered saline solution	NaCl Phosphate buffered saline solution	NaCl Phosphate buffered saline solution
Chromophore osmolarity	Hypotonic	Hypotonic	Hypotonic
Chromophore concentration	0.22% (Paracel 1)	0.22% (Paracel 1)	0.22% (Paracel 1)
	0.25% (Paracel 2)	0.25% (Paracel 2)	0.25% (Paracel 2)
Epithelial rinsing	15 s with BSS	15 s with BSS	15 s with BSS
Light source	New KXL I (Glaukos-Avedro, USA)	New KXL I (Glaukos-Avedro, USA)	New KXL I (Glaukos-Avedro, USA)
Irradiation mode (interval)	Pulsed (1 s on−1 s off)	Pulsed (1 s on−1 s off)	Pulsed (1 s on−1 s off)
Protocol modifications	EFPL M ACXL	EFPL M ACXL	EFPL M ACXL
Protocol abbreviation	PFPL M ACXL	PFPL M ACXL	PFPL M ACXL

The surgical procedure was performed under topical anesthesia with 4% oxybuprocaine chlorhydrate 1.6 mg/0.4 ml drops, applied 5 min before the treatment (one drop per min). After applying a closed-valve eyelid speculum, it involved swiping the epithelial surface with a sponge soaked in 0.25% Paracel 1 solution. This was followed by biphasic (4 min part one and 6 min part two) riboflavin soaking with Paracel 1 (Glaukos-Avedro, Burlington, USA) 0.25% riboflavin solution, dropped every min for 6 min, and Paracel 2 (Glaukos-Avedro, Burlington, USA) 0.22% riboflavin solution, dropped every 30 s for 4 min. Copious irrigation of the corneal surface was performed for 15–20 s with a balanced salt solution (BSS) before starting the UV-A irradiation with the New KXL I UV-A emitter (Glaukos-Avedro, Burlington, USA).

The new Epi-On protocol is a pachymetry-based variable fluence “corneal photodynamic therapy.” It consists of the application of a progressively higher fluence of 7.2 in corneas with baseline pachymetry <420 μm (group 1: 8 eyes) using a UV-A power of 18 mW/cm^2^, 8.6 J/cm^2^ in corneas with baseline pachymetry ≥ 420 μm <460 μm (group 2: 11 patients) using 22 mW/cm2 UV-A power, and 10 J/cm^2^ in patients with baseline pachymetry ≥ 460 μm (group 3: 13 patients) using 26 mW/cm^2^ UV-A power. Overall, an irradiation time of 13 min was maintained in all cases to achieve an overall treatment time of 23 min (10 min of riboflavin soaking plus 13 min of pulsed light 1 s on−1 s off UV-A irradiation), as shown in Table 2. At the end of the procedure, the cornea was medicated with preservative-free netilmicin plus dexamethasone, cyclopentolate, and Ketorolac eye drops plus Carbopol 974P 0.25 % gel. The eye was dressed with a therapeutic bandage soft contact lens, which was kept for 24 h. After therapeutic contact lens removal (the day after), fluorometholone 0.2% eye drops (tapered 4 times/day in the first week, 3 times/day in the second week, and 2 times/day in the third week) were administered in combination with sodium hyaluronate 0.2% lacrimal substitutes for 6 weeks. In case of pain, oral NSAID (10 mg Ketorolac) was prescribed in the first 24 h in combination with Ketorolac eye drops administered qid.

### Statistical analysis

According to the study purpose, follow-up examinations were performed at 24 h, 1 month, 3 months, and 6 months. All patients completed the 6-month follow-up. A two-tailed paired samples *t*-test was performed to compare each baseline measurement with the respective follow-up measurements. Differences with *P* < 0.05 were considered statistically significant. Data were collected and analyzed with PRISM 6.0 GraphPad software (La Jolla, California, USA).

## Results

**Figure 1:**
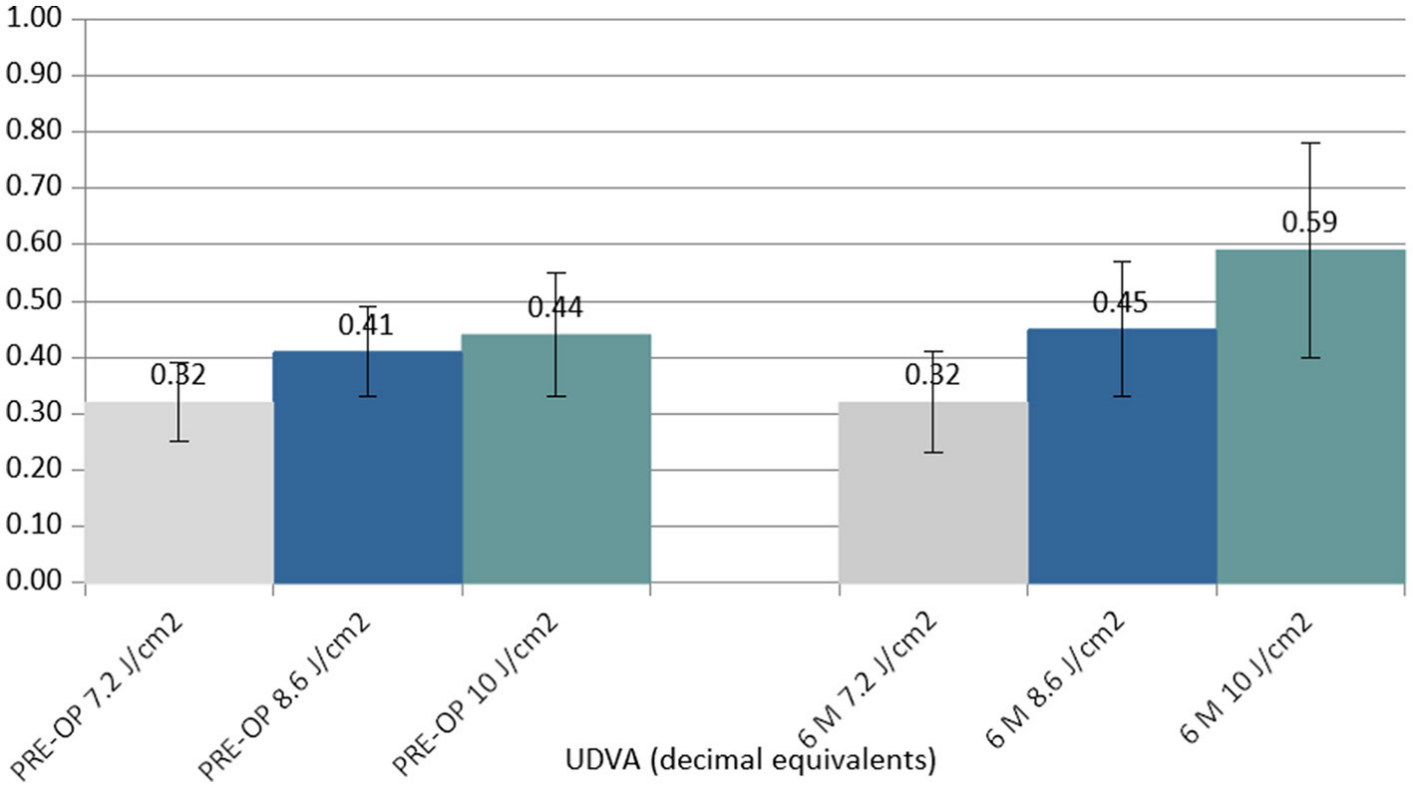
Column chart with preoperative (**Left** cluster) and 6-month postoperative (**Right** cluster) values of uncorrected distance visual acuity (UCVA) expressed in decimal equivalents. In each cluster, the first column (gray) corresponds to group 1 (7.2 J/cm^2^, eight patients), the central column (blue) corresponds to group 2 (8.6 J/cm^2^, 11 patients), and the right column (green) corresponds to group 3 (10.0 J/cm^2^, 13 patients).

Between the preoperative data and the 6-month postoperative control data, the UDVA did not show statistically significant changes in the 7.2 J/cm^2^ (no detectable difference found) and 8.6 J/cm^2^ (Δ +0.04 decimal equivalents) groups, but it showed a statistically significant improvement in the 10.0 J/cm^2^ group (Δ +0.15 decimal equivalents) (Figure 1).

**Figure 2:**
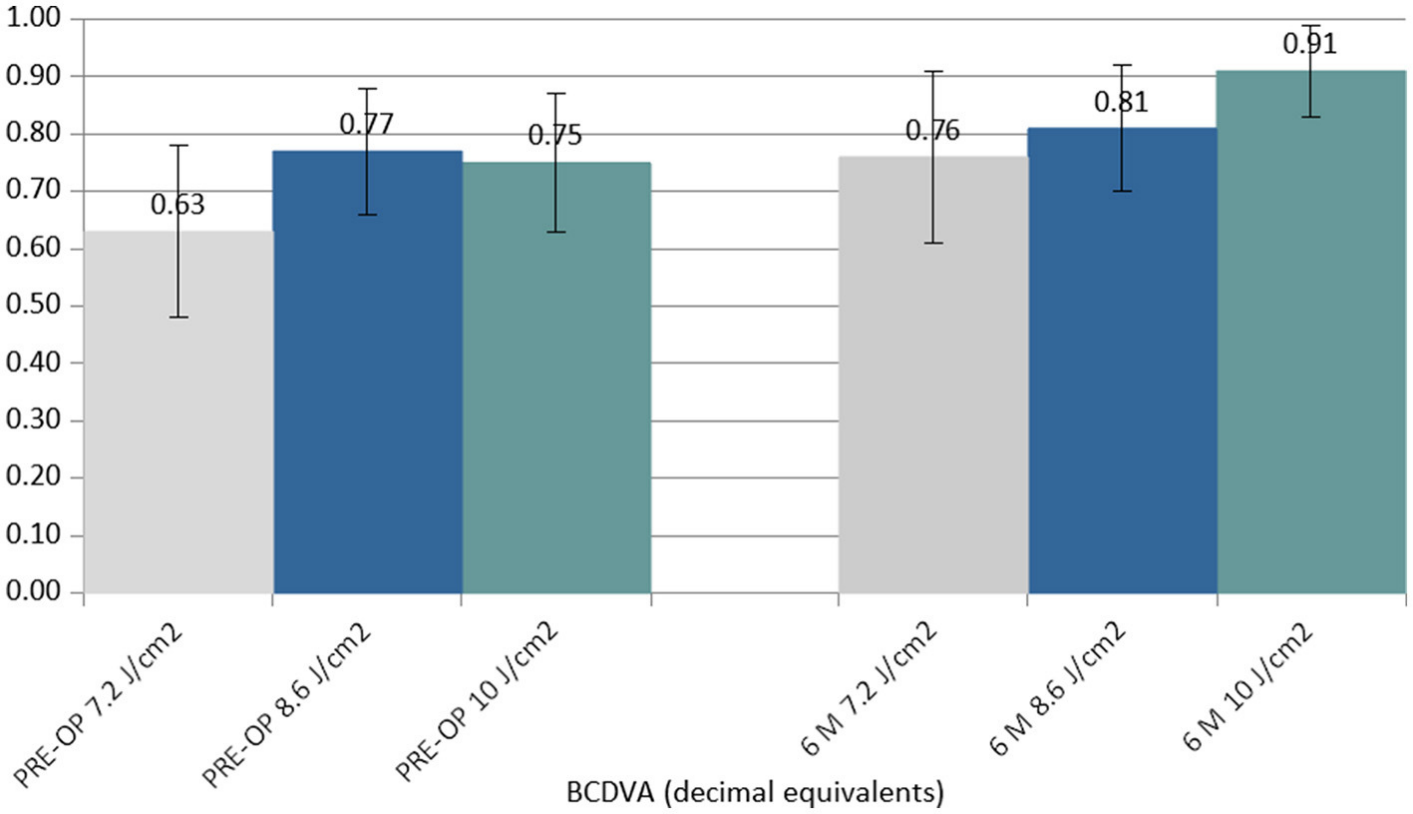
Column chart with preoperative (**Left** cluster) and 6-month postoperative (**Right** cluster) values of best-corrected distance visual acuity (BCDVA) expressed in decimal equivalents. In each cluster, the first column (gray) corresponds to group 1 (7.2 J/cm^2^, eight patients), the central column (blue) corresponds to group 2 (8.6 J/cm^2^, 11 patients), and the right column (green) corresponds to group 3 (10.0 J/cm^2^, 13 patients).

At 6 months postoperatively, the BCDVA did not show statistically significant changes in the 7.2 J/cm^2^ (Δ +0.13 decimal equivalents) and 8.6 J/cm^2^ (Δ +0.04 decimal equivalents) groups, but it showed a statistically significant improvement in the 10.0 J/cm^2^ group (Δ +0.16 decimal equivalents) (Figure 2).

**Figure 3:**
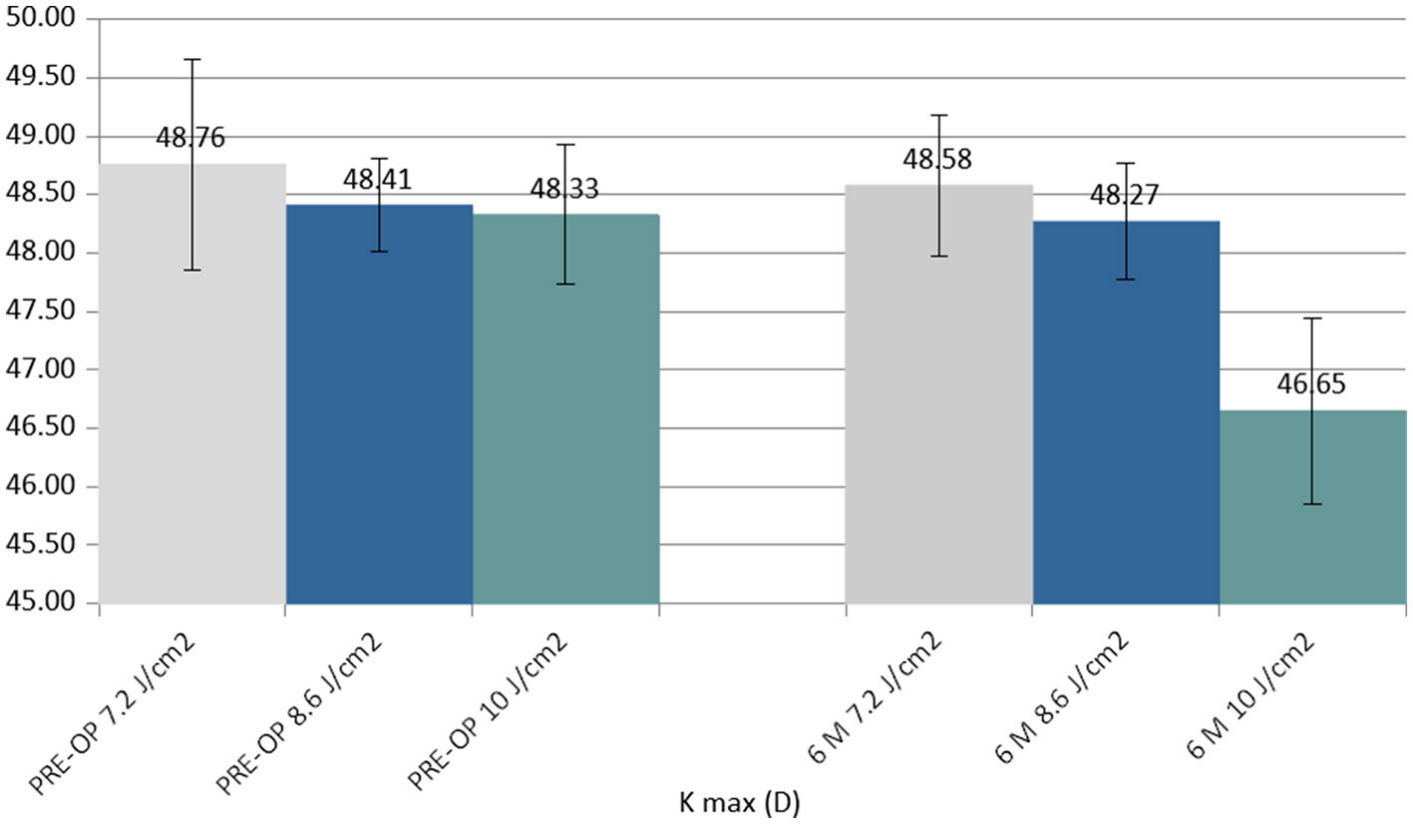
Column chart with preoperative (**Left** cluster) and 6-month postoperative (**Right** cluster) values of maximum curvature simulated K reading (K max), expressed in diopters (D). In each cluster, the first column (gray) corresponds to group 1 (7.2 J/cm^2^, 8 patients), the central column (blue) corresponds to group 2 (8.6 J/cm^2^, 11 patients), and the right column (green) corresponds to group 3 (10.0 J/cm^2^, 13 patients).

K max reduced in all the groups at 6 months postoperatively. The difference between the 7.2 J/cm^2^ and 8.6 J/cm^2^ groups was not statistically significant, whilst the difference between the 10.0 J/cm^2^ group and the other groups was statistically significant (*P* < 0.05) (Figure 3).

**Figure 4:**
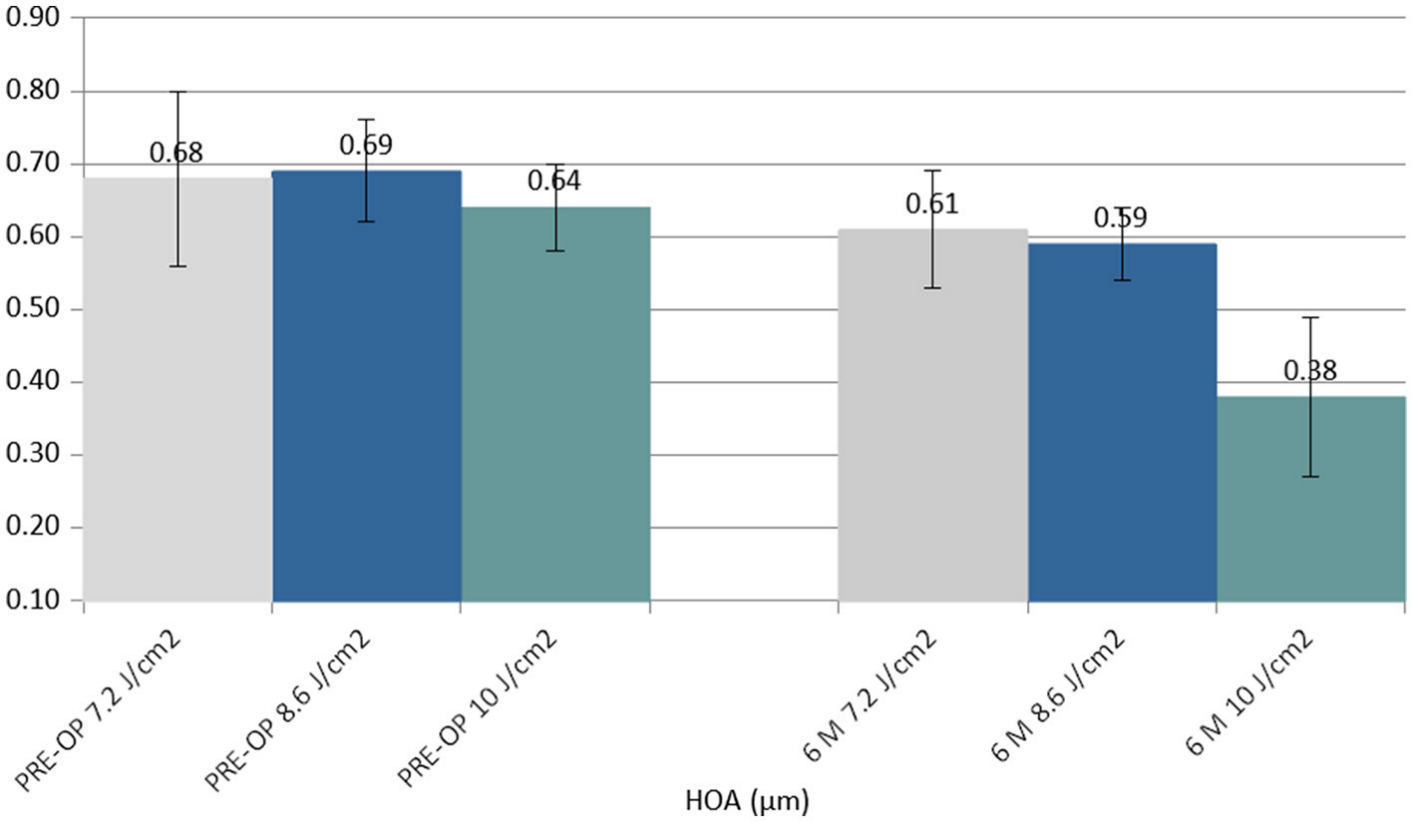
Column chart with preoperative (**Left** cluster) and 6-month postoperative (**Right** cluster) values of coma (high order aberration, HOA), expressed in micrometers (μm). In each cluster, the first column (gray) corresponds to group 1 (7.2 J/cm^2^, eight patients), the central column (blue) corresponds to group 2 (8.6 J/cm^2^, 11 patients), and the right column (green) corresponds to group 3 (10.0 J/cm^2^, 13 patients).

The coma (HOAs) value showed a statistically significant improvement in the 6th month in the 10.0 J/cm^2^ group (Δ−0.26 μm) (Figure 4).

**Figure 5:**
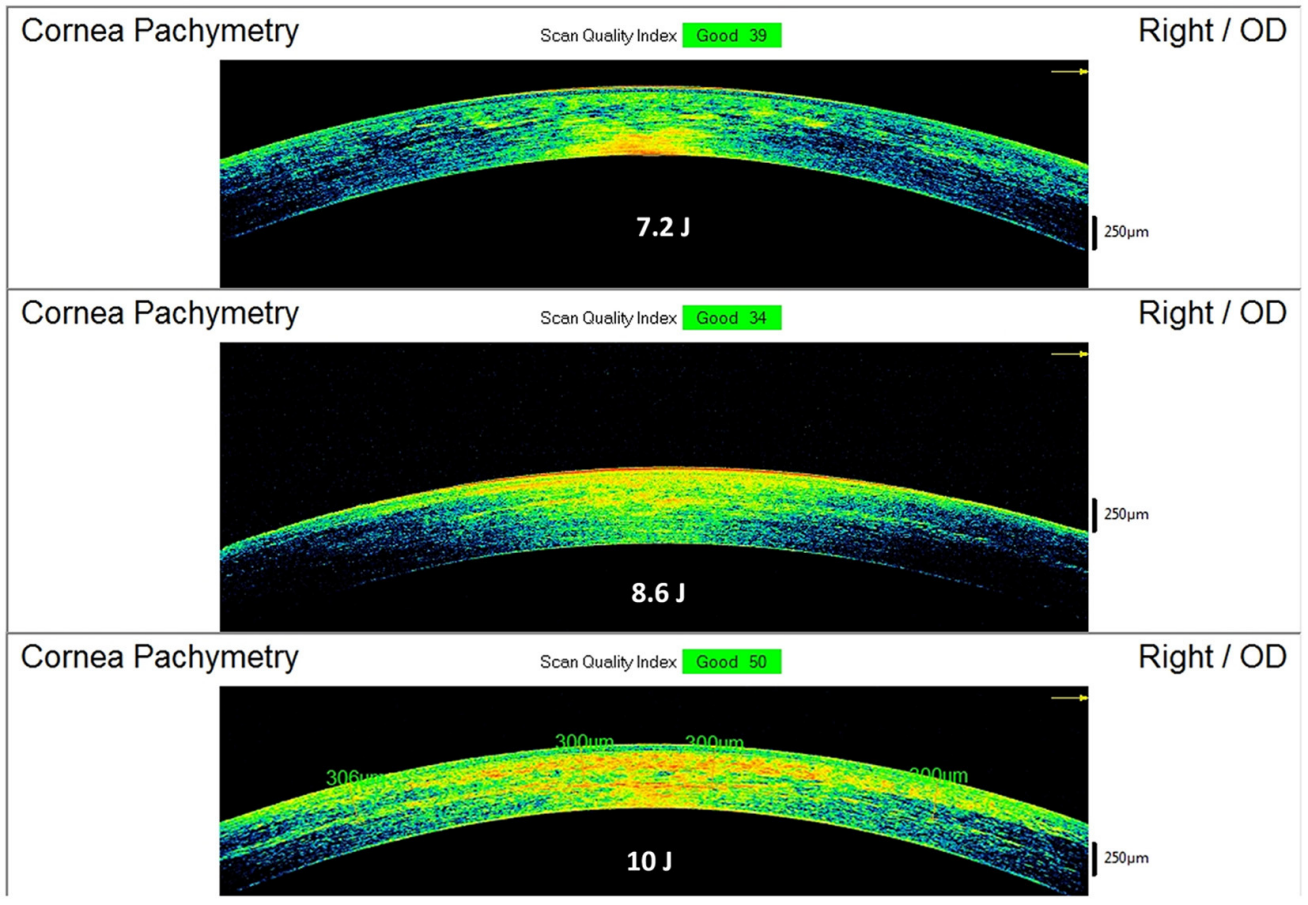
Spectral-domain corneal OCT scans performed 15 days after Progressive Fluence Pulsed Light Epi-On M nomogram (PFPL M Epi-On), showing the depth of the demarcation line and stromal reflectivity. The 7.2 J/cm^2^ group (see example in the **Top** image) had an average demarcation line of 211 ± 19 μm; the 8.6 J/cm^2^ group (see example in the **Middle** image) had an average demarcation line of 245 ± 23 μm; the 10 J/cm^2^ group (see example in the **Bottom** image) had an average demarcation line of 267 ± 21 μm.

The postoperative spectral-domain corneal OCT performed 1 month after the treatments revealed a clear demarcation line with a mean depth of 211 ± 19 μm in group 1, 245 ± 23 μm in group 2, and 267 ± 21 μm in group 3 (Figure 5).

No adverse events, such as haze or infections, were recorded during the follow-up. A punctate epitheliopathy was found in all cases after performing the fluorescein dye test, which resolved after 24–48 h when a soft contact lens bandage and lubricants were applied. According to the visual analog scale (VAS) pain scale, the average value as reported in our series was 2 ± 1, from 0 (no pain) to mild annoying pain (2) in the first 2–4 postoperative hours. The postoperative endothelial count showed no significant variation in all three groups between preoperative and 6-month postoperative data.

## Discussion

This pilot study demonstrated for the first time at the international level the efficacy and safety of a pachymetry-based progressively higher fluence Epi-On in halting KC progression in all treated patients. The treatment helped avoid pain, eliminated the risk of infectious keratitis, prevented persistent stromal haze, and resulted in faster functional recovery for the patients. This method is the ideal candidate as a possible future alternative to Epi-Off CXL in the management of progressive KC. What is even more relevant is the absence of risks and adverse events, which place this method amongst the prophylactic therapies of primary and iatrogenic corneal ectasia as it can be applied purely preventively, even in cases of ectasia that have not yet evolved, to safeguard patients' vision with excellent compliance. The approach also allows for the possibility of simultaneous outpatient interventions, causing minimal postoperative discomfort and avoiding microstructural damage to the ocular surface so that patients may return to their normal activities the day after (9). The high fluence era began in 2016, with studies on customized CXL by Seiler (the first study with 10 J/cm^2^) (11) and Mazzotta (the first study with PiXL at 10 and 15 J/cm^2^) (12). It is known that customized CXL with variable fluence shows multiple demarcation lines; this formed the theoretical basis for the formulation of the “Epi-On M Nomogram” in this study. An increase in UV-A irradiation causes an increase in the density of cross-links in the corneal tissue, even in a low-oxygen environment. Thus, it is apparent that relatively higher fluences help improve stromal strength (9). Therefore, increasing the treatment fluence not only increases the demarcation line depth but also improves corneal resistance to progressive ectasia, which is crucial to Epi-On treatments (9). A higher fluence results in a deeper demarcation line, substantially increasing the treatment volume and the biomechanical power of crosslinking itself (11, 22–27). The clinical and experimental observations presented in the literature and in this paper suggest that, currently, the best practice of Epi-On CXL treatment could be setting a balance between the riboflavin concentration and a higher fluence between 7.2 and 10.0 J/cm^2^ (22–24).

The morphological anterior segment OCT and tomographic observations of the demarcation line depth show evidence of fluence-dependent CXL penetration and biological impact (the higher the fluence, the deeper the demarcation line depth and the deeper the stromal density) (11, 12). This apparently demonstrates that we are moving in the right direction to permanently replace the Epi-Off CXL.

**Figure 6:**
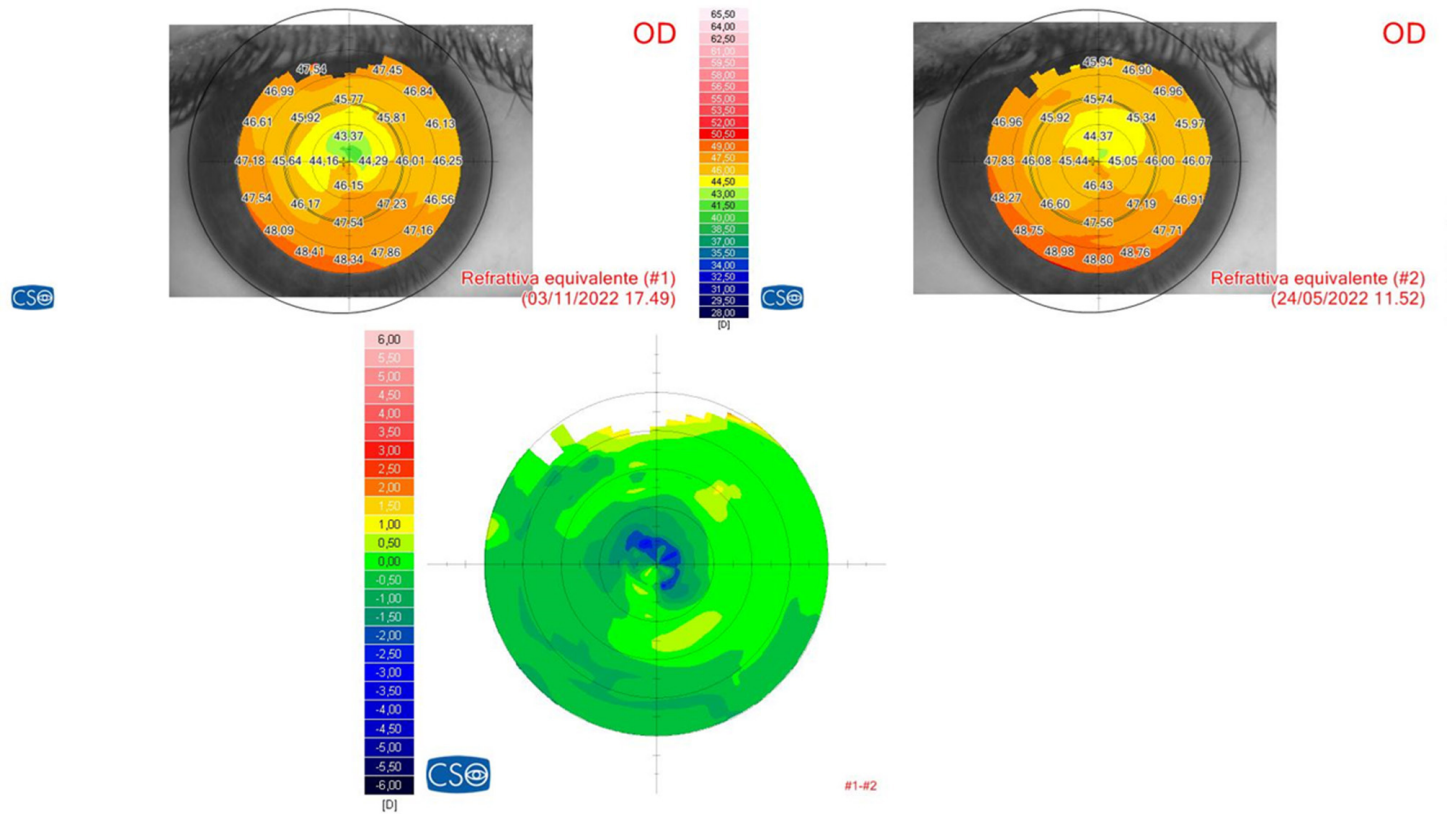
Refractive differential map after Progressive Fluence Pulsed Light Epi-On M nomogram (PFPL M Epi-On). The 6-month follow-up differential tomography after the 10 J/cm^2^ PFPL M Epi-On protocol showed a highly noticeable central corneal flattening.

The preliminary results of this study demonstrate this protocol's ability to penetrate deeply into the stroma without the need to remove the corneal epithelium and to impart a reflectivity to cross-linked tissue, achieving significant corneal flattening that is comparable to that of the Epi-Off treatment, whilst avoiding its side effects, as shown in Figure 6. Moreover, this treatment allows for the possibility of carrying out the procedure in the outpatient mode with less invasiveness and no adjunctive costs (9). Experimental data have proven that higher fluences (irradiation doses) result in a larger increment of corneal stiffness (22–24).

It was previously known that the crosslinking density in the superficial layers ends after reaching saturation and cannot be increased indefinitely (28). Therefore, its overall efficiency attains a homogeneous distribution not only horizontally into the stroma but also in a “depth-dependent curve,” as recently reported in a review study (25). Biochemical investigations have clearly proven that collagen molecules possess many amino acid reactive residues, such as lysine and proline, constituting over 80% of collagen amino acids that are recruited in the strong chemical (aldehydes-mediated) CXL. Contrarily, only a limited amount (<20%) of free reactive residues of aromatic amino acids, such as tyrosine and phenylalanine, are involved in the short-wave UV-mediated CXL (29).

The increase in the effectiveness of Epi-On protocols is due to the combination of several factors, such as high fluence, imbibition with a high concentration of riboflavin carried out by new chemical enhancers, and the use of pulsed UV light (18, 19). These combined factors enhance the anaerobic photodynamic pathways of CXL (8). This has been extensively demonstrated in both iontophoresis (15, 16) and non-iontophoresis (9) treatment protocols, using 7 J/cm^2^ fluence, as well as in Epi-On customized ACXL protocols at variable higher fluences (between 7.2 and 10 J/cm^2^) with supplemental oxygen (10).

The fact that laboratory studies confirm that fluences between 8 and 10 J/cm^2^ determine the best biomechanical result cuts the head off all low-fluence treatments especially if conveyed by transepithelial route (22–24).

In fact, it is known that the antioxidant systems of the corneal epithelium and Bowman's lamina absorb approximately 30% of the UV-A energy at a wavelength of 370 nm (30, 31). Corneal transepithelial photodynamic treatments with low fluence between 2.7 and 3.6 J/cm^2^ with pulsed light cannot prevent the long-term evolution of ectasia due to low CXL density. Additionally, they do not avoid the risk of developing ectasia in the presence of undiagnosed fruste keratoconus (FFKC) due to the obvious limitations outlined above (epithelium-Bowman's lamina complex antioxidants UV-energy absorption and low biomechanical efficacy) (30, 31) and due to high oxygen consumption provided by the epithelium *in situ*. This creates a further negative impact on oxygen kinetics and on the overall efficacy of the Epi-On treatment as a low-fluence corneal CXL photodynamic treatment (LF-CXL-PDT) (32, 33).

What we define as low-fluence crosslinking corneal photodynamic treatment (LF-CXL-PDT) may have a rationale for use in femto-LASIK (34) and corneal refractive lenticule extraction techniques (ReLEx) (35, 36) to prevent idiopathic iatrogenic ectasias. This approach helps avoid an aggressive impact that could negatively affect the corneal stroma, modifying the postoperative refraction stability due to uncontrolled apoptotic corneal flattening and stromal collagen fibril retraction. However, further studies are required to standardize the UV-A irradiation protocols and to evaluate the long-term safety, refractive predictability, and stability of these procedures.

The early evidence of our study will allow surgeons to extend the indications for the photodynamic variable fluence ACXL protocol to increasingly younger patients, potentially to treat developmental ectasias even in the pediatric age, before or after intrastromal refractive surgery procedures (LASIK and ReLEx). It may also allow them to perform bilateral treatments when needed, with much-improved patient compliance and clinical workflow, including the possibility of outpatient treatments conducted on the same day to reduce the burden of time and organization for both the patient and the surgeon. Moreover, this treatment protocol can be repeated if the progression of ectasia restarts during the follow-up, and the advantages of Epi-On procedures can still be retained in terms of excellent compliance and the absence of infectious and uncontrolled corneal scarring (9).

In conclusion, the preliminary results of the study show that PFPL M Epi-On ACXL halted KC progression in young-adult patients and demonstrated increased efficacy closer to the standard Epi-Off CXL. The increase in efficacy was directly correlated with higher fluences, and significantly better results were obtained by adopting a fluence of 10 J/cm^2^.

Further studies with longer follow-up time, larger patient cohorts, and with the implementation of additional parameters are necessary to improve the efficacy of epithelium-preserving CXL protocols and make them highly effective, more accessible, and feasible in outpatient modality.

## Data availability statement

The raw data supporting the conclusions of this article will be made available by the authors, without undue reservation.

## Ethics statement

The treatment protocol was approved by the Institutional Review Board of the Siena Crosslinking Center (Code: PFPL.EPION 2.0). The patients/participants provided their written informed consent to participate in this study.

## Author contributions

Conceptualization, writing—original draft, writing—review and editing, methodology, and investigation: CM and MF. Supervision: AP. Data curation: CM and AP. All authors contributed to the article and approved the submitted version.
